# Defining ELISpot cut-offs from unreplicated test and control wells

**DOI:** 10.1016/j.jim.2013.02.014

**Published:** 2013-06-28

**Authors:** Neal Alexander, Annette Fox, Vu Thi Kim Lien, Tao Dong, Laurel Yong-Hwa Lee, Nguyen Le Khanh Hang, Le Quynh Mai, Peter Horby

**Affiliations:** a London School of Hygiene and Tropical Medicine, Keppel Street, London WC1E 7HT, United Kingdom; b Oxford University Clinical Research Unit, Wellcome Trust Major Overseas Programme, Hanoi, Vietnam; c Centre for Tropical Medicine, Nuffield Department of Clinical Medicine, University of Oxford, Oxford OX3 7LJ, UK; d National Institute for Hygiene and Epidemiology, 1 Yersin Street, Hanoi, Vietnam; e MRC Human Immunology Unit, Weatherall Institute of Molecular Medicine, John Radcliffe Hospital, Oxford, United Kingdom

**Keywords:** DMSO, dimethyl sulphoxide, ELISpot, enzyme-linked immunospot, ECDF, empirical cumulative distribution function, IAVI, International AIDS Vaccine Initiative, PBMC, peripheral blood mononuclear cell, PHA, phytohemagglutinin, ROC, receiver operating characteristic, SFU, spot forming units, ELISpot, Cut-off, Standardized

## Abstract

In the absence of replication of wells, empirical criteria for enzyme-linked immunospot (ELISpot) positivity use fixed differences or ratios between spot forming units (SFU) counts between test and control. We propose an alternative approach which first identifies the optimally variance-stabilizing transformation of the SFU counts, based on the Bland–Altman plot of the test and control wells. The second step is to derive a positivity threshold from the difference in between-plate distribution functions of the transformed test and control SFU counts. This method is illustrated using 1309 assay results from a cohort study of influenza in Vietnam in which some, but not all, of the peptide pools have clear tendencies for SFU counts to be higher in test than control wells.

## 1 Introduction

Since it was first described in 1983, the enzyme-linked immunospot (ELISpot) assay has become a widely used method for the detection of antigen-specific cytokine-secreting T cells (Czerkinsky et al., 1983; Versteegen et al., 1988), and is now a standard assay for measuring the cell-mediated immune response to vaccines in clinical trials. The requirement for immunological assays used in vaccine trials to be rigorously validated has resulted in much work to maximize the sensitivity and specificity of ELISpot assays, ensure their reproducibility, minimize inter-laboratory and inter-operator variability and to automate and standardize the counting of the spot forming units (SFU) (Vaquerano et al., 1998; Schmittel et al., 2000; Mwau et al., 2002; Janetzki et al., 2004, 2005, 2008; Cox et al., 2005; Lehmann, 2005; Samri et al., 2006; Maecker et al., 2008). However, criteria for defining a positive response have been subject to considerable debate and controversy (Mwau et al., 2002; Hudgens et al., 2004; Jamieson et al., 2006; Jeffries et al., 2006; Moodie et al., 2010; Slota et al., 2011).

Since the spot counts in the negative control wells, which contain no stimulating analyte, are predictive of the background count in the wells that contain peptide (the experimental wells) it makes sense to use comparisons between the negative control and the experimental wells to define responsiveness (Hudgens et al., 2004). This approach is further supported by the variability in background spot counts between and within laboratories and individuals, and even within samples depending on their handling, which mean that universal cut-offs are generally not credible (Hudgens et al., 2004; Cox et al., 2005). One commonly used technique to define a positive or negative response is to consider a well positive if it contains a pre-defined number of SFU above the count in the negative control well, with values of 10–50 SFU/10^6^ PBMC often being used (Schölvinck et al., 2004). This method has the disadvantage of a higher false positive probability in plates with high background, since a chance variation of, for example, 10 spots is more likely with high counts than low counts. A common alternative is to consider a well positive if its number of SFU is above a pre-defined multiple of the control, i.e. a criterion based on a ratio rather than a difference. This has the opposite disadvantage: higher false positive probability in plates with *low* background counts. For example, if the criterion is a four-fold ratio, and the negative control has two spots, an experimental well will be considered positive if it has ≥ eight spots, and this is much more likely to occur by chance than a value of 800 spots where the control well has 200. These considerations have led many groups to apply a combination of absolute and fold difference (Larsson et al., 1999; Russell et al., 2003; Jeffries et al., 2006). For example, the T-SPOT manual recommends a difference of at least 6 if the negative control has 5 or fewer spots, and a ratio of at least 2 when it has 6 or more (Oxford Immunotec, 2006). Additionally, a threshold value (e.g. at least 11 SFU/10^6^ PBMC in the experimental well) is also sometimes applied to provide a threshold of responsiveness that is considered to have biological significance. Similarly, an upper limit on the number of spots in the negative control well may be imposed, e.g. 10 in the case of T-SPOT and IAVI (International AIDS Vaccine Initiative)(Gill et al., 2010). These cut-offs and thresholds are often defined with reference to ELISpot responses in a known negative population and are therefore often referred to as *empirical* methods (Moodie et al., 2006). By contrast, *statistical* methods have been developed which use the variation between replicate control wells to define positivity thresholds (Hudgens et al., 2004; Moodie et al., 2006). However, when a wide range of peptides is being examined it may be impractical to include replication of the peptide and negative control wells. In the current paper we develop a positivity criterion for such plate layouts, in the context of a study of cell mediated immunological response to influenza.

## 2 Methods and results

We present a method which uses within-plate differences between test and control wells, and a positivity threshold based on their statistical distribution over plates. The method relies on the principle that pools can only be reliably declared positive when the test counts tend to be larger than the negative control ones. The method is illustrated using data from a cohort study in Vietnam (Horby et al., 2012).

### 2.1 Study population

The cohort study included 932 individuals aged between 5 and 90 years. PBMC samples were taken to measure the prevalence of T-cell responsiveness to seasonal and avian influenza peptides in order to determine the protective effect of pre-existing T-cell responses. Institutional review boards in the United Kingdom and Vietnam approved the study and all subjects provided written informed consent.

### 2.2 ELISpot assay

#### 2.2.1 Antigens

The complete proteome of H3N2 (A/NewYork/388/2005), the haemagglutinin and neuraminidase of H1N1 (A/Hong Kong/1134/98 and A/New York/228/2003) and the haemagglutinin of H5N1 (A/Vietnam/CL26/2004) were represented as 14–20 amino acid peptides overlapping by 10 amino acids. Peptides representing each protein were tested as either 1 or 2 pools containing between 24 and 52 peptides, giving a total of 20 pools. The final culture concentration of individual peptides was 2–3 μg/ml. Phytohemagglutinin (Sigma-Aldrich) was used at 10 μg/ml.

#### 2.2.2 PBMC preparation and IFN-γ ELISpot

Heparinized venous blood was received within eight hours of collection and immediately overlayed onto Lymphoprep then centrifuged to isolate PBMCs. PBMCs were either tested in ELISpot immediately or cryopreserved in fetal calf serum containing 10% DMSO. ELISpot was performed according to published protocols (Lalvani et al., 1997). In brief, 250,000 PBMC per well were incubated with peptide pools, PHA or media-only (negative control) overnight. ELISpot plates were scanned using a Cellular Technology Ltd. Series 3A Analyzer. Spots were then counted using ImmunoSpot 3.1 software. Spot definition settings were as follows: sensitivity 170; minimum spot size 0.0142 mm^2^; maximum spot size 0.4399 mm^2^; oversized spots estimated; spot separation 1.00; diffuse spot process on; diffuseness 20; gradient off; overdeveloped area handling active; background balance on; background balance 30; fill holes off. Audit spots was set ‘on’ such that automated counting was subject to manual review whereby areas selected automatically could be de-selected if they appeared to be something other than a spot from IFN-γ release. PHA wells were counted using more sensitive settings. Spot forming unit (SFU) counts were automatically transferred from an automated ELISpot counter (Cellular Technology Limited) to a Microsoft Access database, resulting in 1309 records. Of these, 758 were tested immediately and 551 cryopreserved. We present analysis of all samples irrespective of this status, although supplementary figures show that test SFU counts exceeded those of control more strongly in those samples processed immediately.

### 2.3 Statistical methods

The approach is to first identify a suitable data transformation and then, where feasible, choose a threshold value to define positive wells. This will be illustrated by two of the H1N1 pools from the above study.

As mentioned above, thresholds based on differences between spot counts tend to result in false positive at high values, but those based on ratios — or, equivalently, differences on the log scale — result in the opposite problem. This is because the variance of the untransformed counts increases with the mean value, and this trend is reversed by the logarithmic transformation. The property of the variance changing with the mean — whether increasing or decreasing — is known as heteroscedasticity. Since the logarithmic transformation can be seen as the limit of a series of power transformations (Tukey, 1957) — e.g. square root, cube root, and so on — we seek the power which minimizes heteroscedasticity. More specifically, for each power we plot the difference of the transformed values against their average — a Bland & Altman plot (Bland and Altman, 1986) — and minimize the chi-squared statistic of a test for heteroscedasticity in the corresponding regression (Breusch and Pagan, 1979). We also used the studentized version of the test, which is more robust to non-Gaussian variation (Koenker, 1981), and the results remained identical to at least two decimal places. Once the power transformation has been selected, the regression is not used further.

**Fig. 1:**
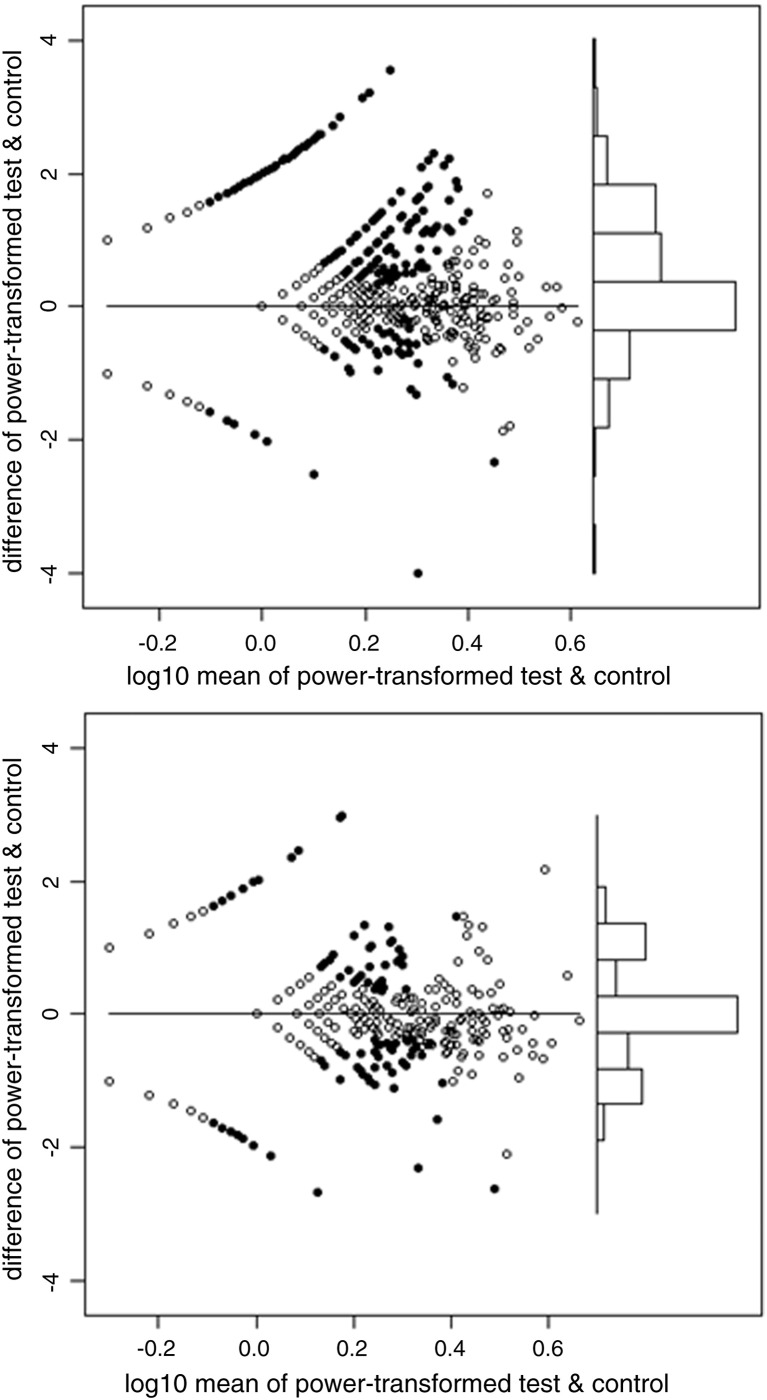
Bland–Altman plots for the power-transformed counts for haemagglutinin (upper panel) and neuraminidase (lower panel). Each vertical axis is the difference between the transformed test and control values. Each horizontal axis is the average of the transformed values, on a log scale. In the main part of each plot, to the left, the points are arranged in curved lines because of the original data are integers (whole numbers). In particular, the curved line closest to the top left corner of each plot contains samples with a zero control result (but varying test counts), and the line closest to the bottom left corner contains those with a zero test result (but varying control counts). The solid points are those which would be positive on the T-SPOT criteria, either for test minus control, or ‘pseudo-positives’ in which control minus test would meet those same criteria. The power which minimizes the relation between the variance and mean of the differences in transformed counts is 0.26 for haemagglutinin and 0.27 for neuraminidase. Towards the right of each plot there is a histogram of the differences between the transformed values of test and control, using the same vertical axis. For haemagglutinin, but not for neuraminidase, there is a visible pattern of positives predominating over pseudo-positives.

For the 20 pools, the selected powers ranged from 0.23 to 0.31, mean 0.27. In other words, the optimal transformations were close to fourth root (power = 1/4). Fig. 1 shows the Bland and Altman plots for the first haemagglutinin pool, and the second neuraminidase pool. These plots also show i) the test wells positive on the T-SPOT criteria (see Introduction), and ii) the control wells which would have been positive on the same criteria, had the test and control status been reversed, hereafter referred to as pseudo-positive. For haemagglutinin, the T-SPOT-positive test wells greatly outnumber the pseudo-positive control wells (247:46), but this is not the case for neuraminidase (58:59). By quartile on the horizontal axis, the proportions positive on the T-SPOT criteria are: 0, 23, 26 and 32% for haemagglutinin and 0, 0, 6 and 16% for neuraminidase.

To select a threshold value for defining positive wells, we use the principle that test minus control values should, on average, be larger than control minus test. Otherwise, there is no evidence of a ‘signal’ over the ‘noise’ of control variation, and any positivity threshold is dubious. To select the threshold we compare the empirical cumulative distribution functions (ECDFs) of i) test–control for those plates with test > control and ii) control–test for those with control > test. The ECDF of a sample is simply the proportion of the data points which lie at or below a given value. The difference between ECDFs can be used to discriminate between a mixture of two distributions. In particular, the value which maximizes the difference in ECDFs also maximizes the probability of correct classification (Stoller, 1954). Hence, for the current purpose, we choose the threshold to be the value which maximizes the difference between the above two ECDFs. Pools whose difference over control exceeds this value are declared positive. In principle it is possible for this maximum difference in ECDFs to occur at more than one value on the horizontal axis. Hence we define the threshold, more precisely, to be the lowest such value on the horizontal axis.

**Fig. 2:**
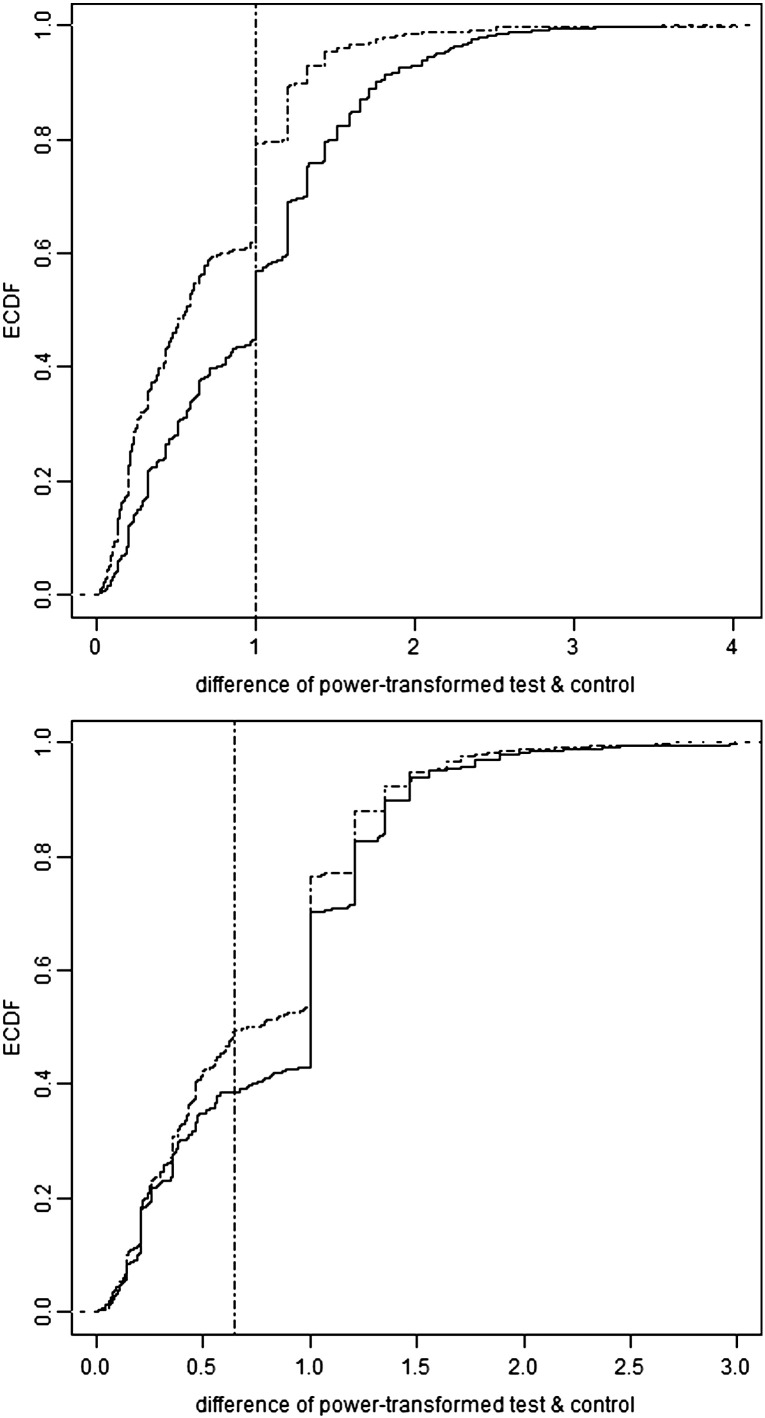
Empirical cumulative distribution functions (ECDF, vertical axis) for the power-transformed positive differences (horizontal axis) of i) test over control (solid line) and ii) control over test (dashed line). The upper panel shows haemagglutinin and the lower one neuraminidase. Each ECDF is the proportion of data points below the corresponding value on the horizontal axis. Each vertical line indicates the greatest separation of the two ECDFs. For haemagglutinin, the ECDFs are further apart than for neuraminidase, indicating a greater tendency for the haemagglutinin test wells to have more SFU than the negative control.

This is shown in Fig. 2 for the two selected pools. Greater data values shift the ECDF to the right, making it lower at any given point on the horizontal axis. For haemagglutinin, the ECDFs of test-minus-control and control-minus-test are much more widely separated than for neuraminidase. For haemagglutinin, the maximum difference in ECDFs is 0.22 and occurs at a transformed test-minus-control value of 1 (i.e. a value greater than 1 is considered positive). For neuraminidase the maximum difference in ECDFs is 0.11 and occurs at a test-minus-control value of 0.64. Applying these threshold values to Fig. 1 gives 291 positive test wells and 63 pseudo-positive control wells for haemagglutinin. The corresponding numbers for neuraminidase are much closer — 222 and 204 — suggesting that reliable discrimination is not possible for neuraminidase. By quartile of the transformed mean, the proportions positive for haemagglutinin are: 0, 68, 13 and 15%, and for neuraminidase are 22, 50, 12 and 11%.

The maximum difference between the two ECDFs is also used by the Kolmogorov–Smirnov test for differences between distributions. A large p value from this test would again suggest that reliable identification of positive samples is not possible, although the converse is not necessarily true. In other words, the p value being less than 5%, for example, does not imply that reliable identification will be possible. Rather, the hypothesis test screens out examples for which no reliable identification can be expected (Armitage et al., 2001, page 472). Over all 20 pools, the p values ranged from 2 × 10^− 16^ to 0.67, those for haemagglutinin and neuraminidase being 2 × 10^− 9^ and 0.02 respectively. Hence for some pools there is no tendency for test to exceed control, as opposed to the other way round, and in such cases trying to assign a threshold would be futile.

This analysis can be expressed in terms of the probability of correctly identifying which pool is test and which is control, when this status is unknown. Suppose we have i) one person's test and control results *x* and *y* (possibly on a transformed scale), *x* being the larger, but without knowing whether *x* or *y* is test, and ii) the distribution of previous test-minus-control values (with the experimental conditions known). We expect larger values to result from the test condition, so suppose our rule is to conclude that *x* is from the test condition if it exceeds the smaller one by more than a value *k*. The conditional probability that *x* is the test sample, given that *x* − *y > k*, is

$$\begin{array}{l} \mathrm{Prob}\left(x\;\mathrm{is}\;\mathrm{test}\left|x-y>k\right)\right)=\frac{\mathrm{Prob}\left(x\;\mathrm{is}\;\mathrm{test}\;\&\;x-y>k\right)}{\mathrm{Pr}\mathrm{ob}\left(x-y>k\right)}\\ \;=\frac{\mathrm{Pr}\mathrm{ob}\left(x\;\mathrm{is}\;\mathrm{test}\;\&\;x-y>k\right)}{\mathrm{Pr}\mathrm{ob}\left(x\;\mathrm{is}\;\mathrm{test}\;\&x-y>k\right)+\mathrm{Pr}\mathrm{ob}\left(x\;\mathrm{is}\;\mathrm{control}\&\;x-y>k\right)} \end{array}$$

**Fig. 3:**
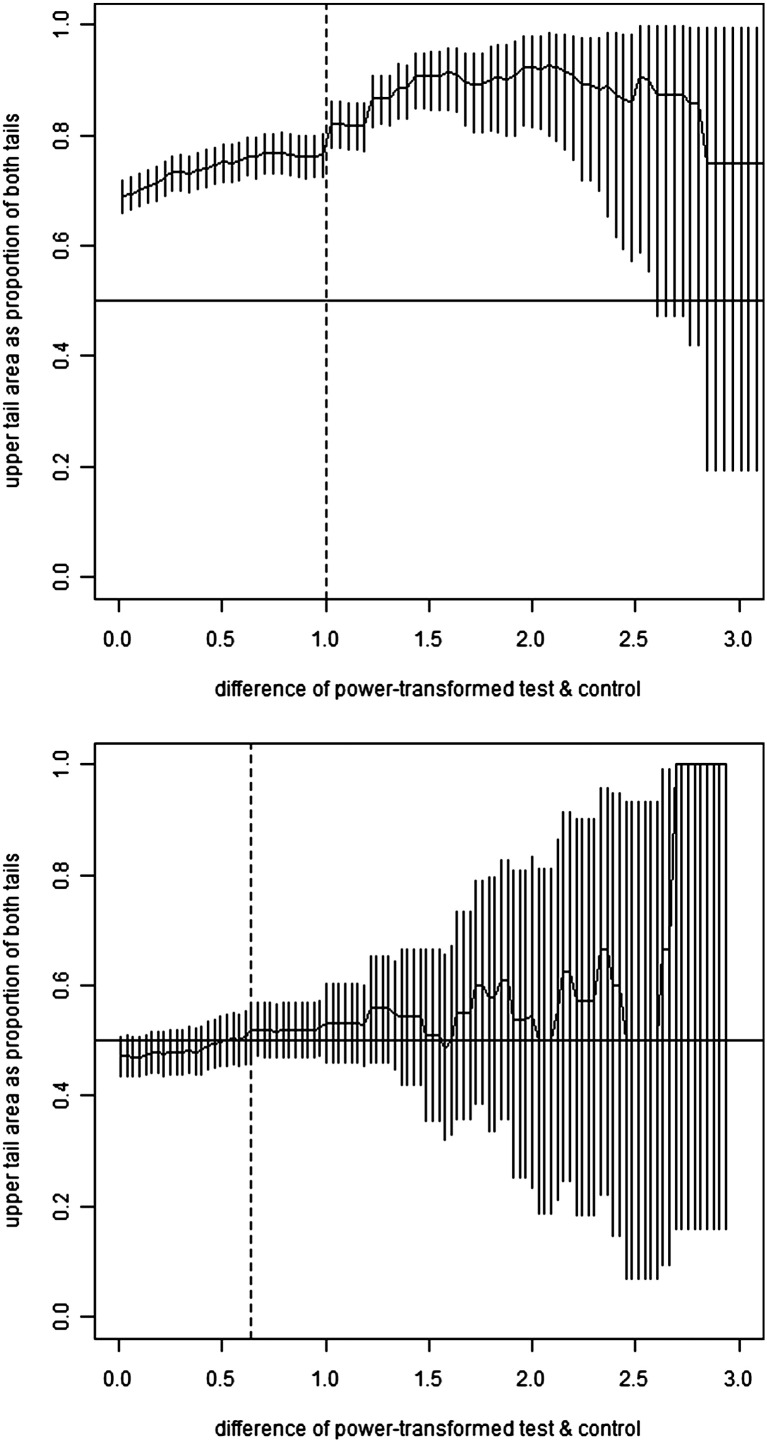
For each panel (upper haemagglutinin, lower neuraminidase), the horizontal axis is the difference between the power-transformed test and control count values. For each such value, the vertical axis shows the corresponding tail probability, i.e. the proportion of the upper and lower tails which is found in the upper tail. For example a proportion of 75% means that the upper tail (test exceeding control by a given amount) has three times as many data points as the lower tail (control exceeding test by that amount). Values near to 50% indicate control SFU counts being, on average, about as high as test. The solid vertical lines are the 95% exact binomial confidence interval for each proportion. These become wide at high values because they are based on few data points. The dashed vertical line on each figure are at the same values as the corresponding panel of Fig. 2. Haemagglutinin, but not neuraminidase, shows a clear ‘signal’ of test over control.

This last expression is the area of the upper tail of the distribution (above a test-minus-control value of *k*) divided by the sum of the upper and lower tails (above *k* or below −*k*). If the control value rarely exceeds the test by *k*, then this probability will be high. This argument is applied to the cohort data in Fig. 3. For haemagglutinin, the test value is likely to exceed control, for a wide range of threshold values. For neuraminidase, however, the control value is about as likely to exceed test as the other way round.

Results from simulated data confirm that the proportion of samples identified as positive increases with the excedent test mean over the control mean (see Supplementary Material). These results also suggest that the current approach may be conservative in identifying positives. There is also a high degree of variation in the estimated proportion positive, which to some extent results from the high variation in the input data. All analysis uses R version 2.11, and the custom-written functions are also included as supplementary material.

## 3 Discussion

Replication of ELISpot test and control wells has been recommended (Moodie et al., 2010) although it reduces the number of proteins that can be tested for given resources. Existing statistical methods utilize this replication to define positivity criteria objectively based on within-plate, between-replicate, variation (Moodie et al., 2012). In the absence of replication, the current approach relies on between-plate variation in a sizable dataset from a given population. The principle is that positivity should tend to give test wells larger counts than control wells.

One problem with existing empirical cut-offs is that large absolute differences are likely to happen by chance when spot counts are high. Log transformation reverses the problem because large fold changes from control can occur by chance at low spot counts. In statistical terms, the original and transformed datasets both have heteroscedasticity, i.e. variance associated with the mean. One solution is to use a transformation which is less strong than the logarithm. The square root transformation may suffice, for example, when the same parasite slide is read twice. This corresponds to the theoretical minimum variation, described by the Poisson distribution of homogeneous counts (Alexander et al., 2007). The current approach selects the power transformation which minimizes heteroscedasticity in the Bland & Altman plot. All of the pools in the example dataset were found to have optimal powers close to ¼, i.e. fourth root transformation, which is between the square root and logarithm in strength.

It was notable that some protein test pools had little or no tendency to exceed the negative (medium) control in terms of spot count. Seeking positive samples is quixotic in these circumstances. In particular, applying existing empirical criteria to such pools, the number of test wells declared positive barely exceeds the number of control wells which would have been declared positive, had the test/control status been reversed in the analysis. When there is a tendency for the differences of test over control to exceed those of control over test, a positivity cutoff can be chosen by comparing their empirical distribution functions (ECDFs), by analogy with non-parametric discrimination (Stoller, 1954). The value corresponding to the maximum difference between the ECDFs gives the greatest probability of successful classification. In practice, however, false negative and false positive errors may not have equal importance, which would suggest increasing or decreasing the cut-off. This kind of calibration, e.g. by receiver operating characteristic (ROC) curve, would require independent identification of true positive and negative individuals. For influenza it is difficult to identify unexposed people from whom to prepare negative sera for this purpose. Such an approach should be more feasible for other infections such as HIV and tuberculosis.

## 4 Conclusions

For situations in which replication of wells is infeasible, we highlight problems with positivity criteria based on fixed differences or ratios between test and control wells, which are known as empirical methods. In our example dataset from a large cohort study, we show that some peptide pools can often be positive on such empirical criteria, while having little or no elevation in SFU over the negative control. We propose an alternative approach which uses within-plate differences between test and control wells, and a positivity threshold based on their statistical distribution over plates.

The following are the supplementary data related to this article.

**Supplementary Fig. 1:**
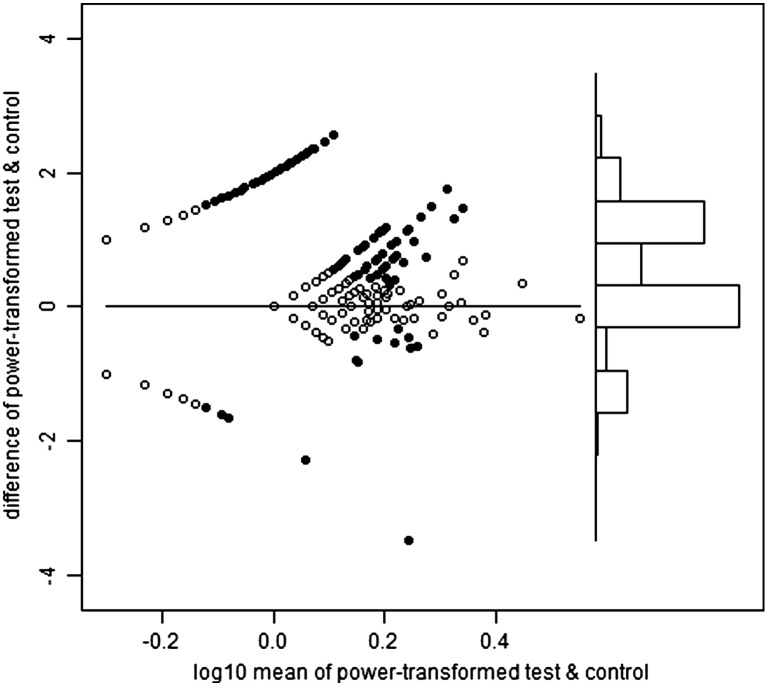
Haemagglutinin Fresh.

**Supplementary Fig. 2:**
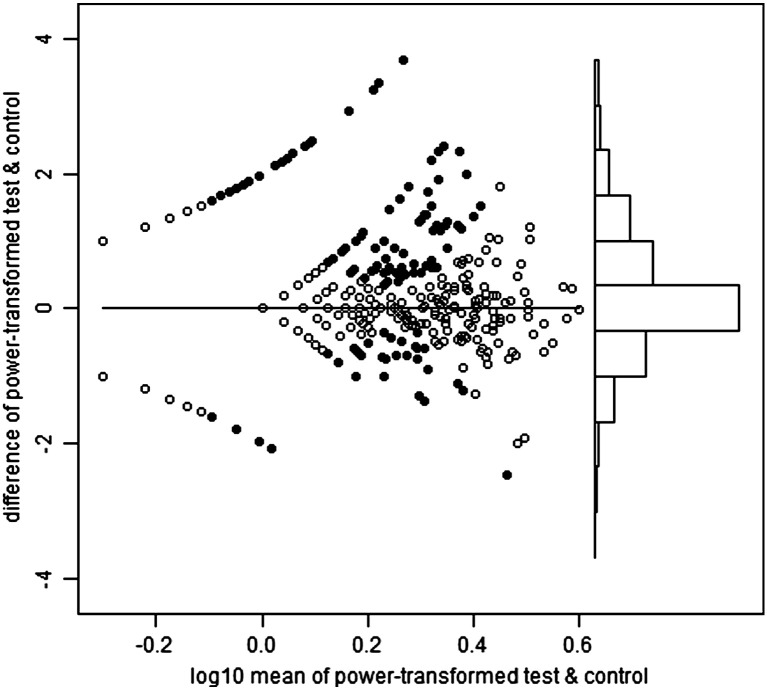
Haemagglutinin Frozen.

**Supplementary Fig. 3:**
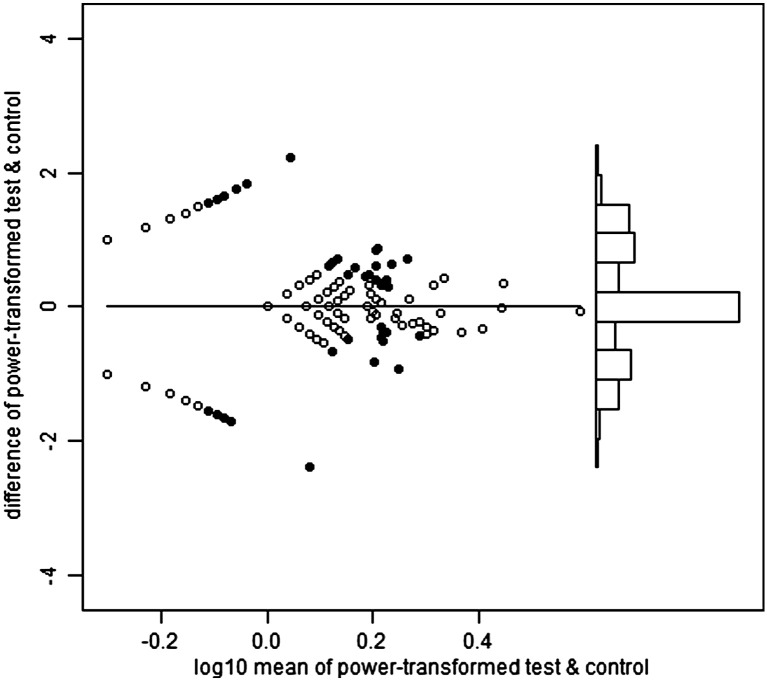
Neuraminidase Fresh.

**Supplementary Fig. 4:**
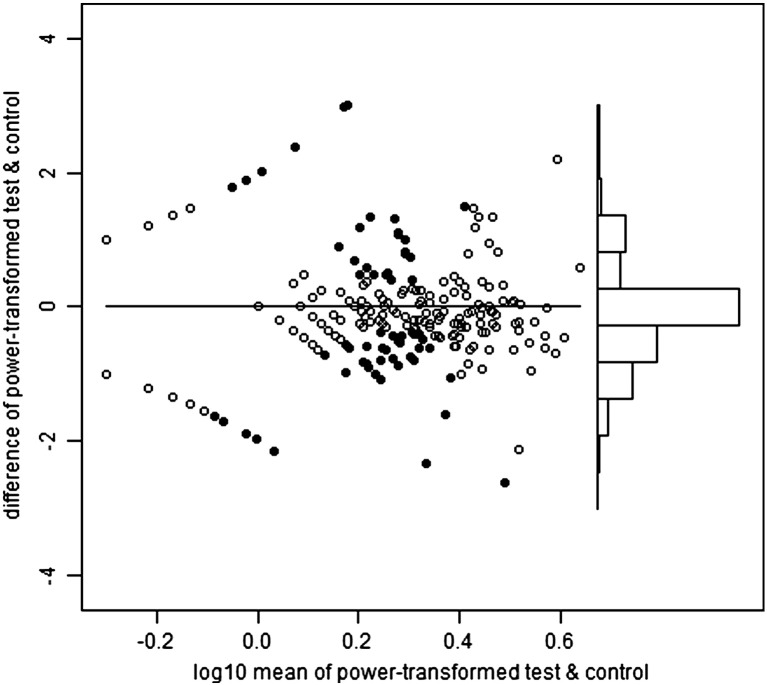
Neuraminidase Frozen.

Supplementary Fig. 5 Results from simulated data.

Supplementary Data Application to Simulated Data.
