# Supplementary figures and images for: Defining ELISpot cut-offs from unreplicated test and control wells
Source: J Immunol Methods. 2013 Jun 28;392(1-2):57–62. doi: 10.1016/j.jim.2013.02.014 (PMC3657161; doi:10.1016/j.jim.2013.02.014)

inferred proportion positive

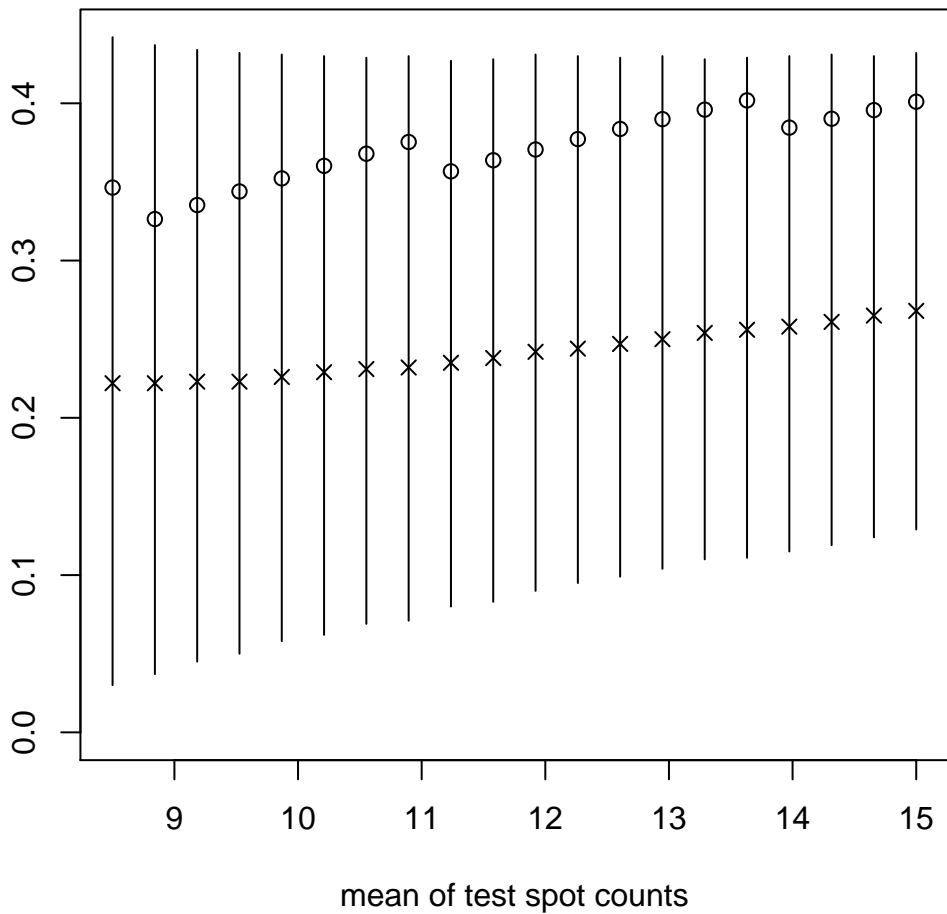

Supplement: Supplementary Fig. 5 — Results from simulated data. [file mmc1.pdf]
