# Supplementary material for: Defining ELISpot cut-offs from unreplicated test and control wells
Source: J Immunol Methods. 2013 Jun 28;392(1-2):57–62. doi: 10.1016/j.jim.2013.02.014 (PMC3657161; doi:10.1016/j.jim.2013.02.014)
Supplement: Supplementary Data — Application to Simulated Data. [file mmc2.docx]

Supplementary Material

**Application to Simulated Data**

To characterize the performance of the proposed method, we simulate *n* pairs of control and test results. The control results are assumed to follow negative binomial distribution with mean *μ*_0_ and dispersion parameter *k*. The test results are assumed to follow a similar, independent, distribution but with *μ*_1_>*μ*_0_.

By construction there is no systematic between-pair variation, such as between plate variation which is the reason for each test well to be compared with its corresponding control. Since the proposed method does not assume knowledge of any true positives, we do not postulate any mechanism or distribution of true positive status at the individual level in the simulated data. Rather we rely on the lack of between-pair variation to again apply non-parametric discrimination based on the ECDFs (empirical cumulative distribution functions) of the test and control results. (Since we do expect between-pair variation in practice, this would not be a good option for real data.) Then we compare the resulting proportion positive with that from application of the proposed method based on difference in transformed values.

Supplementary Figure 5 shows the simulation results for *n*=1000, *k*=0.5, *μ*_0_=7.5 and *μ*_1_ between 8.5 and 15 (horizontal axis). Fifteen thousand datasets were simulated. The vertical axis shows the estimated proportion positive. The ° symbols show the value based on the population ECDFs of the test and control data, which are not subject to sampling variation. The vertical lines show the 95% range, over the simulated datasets, of the proportion positive based on the proposed method, with × showing the median. The results are interpreted in the main text.
